# The Epidemics in Russia and North Germany

**Published:** 1865-05

**Authors:** 


### THE EPIDEMICS IN RUSSIA AND NORTH GERMANY. REPORT OF THE MEDICAL OFFICER OF THE BRITISH PRIVY COUNCIL.

[In the preceding number of the EXAMINER, we suggested that the epidemic in Russia was only a form of typhus. The following is from high authority:—ED.]

Mr. Simon, the medical officer of the privy council of the Queen of England, has reported to Lord Granville the result of the official inquiries which have been made, respecting the epidemic in Russia, and the disease which prevails in North Germany. With respect to the former, he says:---

"As regards St. Petersburg, the epidemic which is prevailing in that city consists of two forms of fever, which are known in this country as relapsing fever and typhus. Of relapsing fever (which, also, from the circumstances under which it prevails, is familiarly known by the name of famine fever), we have had no large experience in this country since the years 1846–48, when, in consequence of the Irish distress, the disease first became epidemic in Ireland, and next raged in Liverpool and many other of our chief towns, to which its contagion was brought by Irish immigrants. Typhus, on the other hand, is probably never absent from among us.

"During the last two or three years, there has been an almost unprecedented amount of it in London, and, as your lordship is aware, inquiries relating to very large epidemics of it in Liverpool, Greenock, and Bristol, have, during the last few months, been required of the medical department of the council office. In times when relapsing fever prevails—and always they are times of national scarcity—typhus always, or nearly always coëxists with it. It was so in Liverpool in 1847, it is so at St. Petersburg at present. The mixed epidemic, wherever it occurs, testifies to the miserable state of a starving and overcrowded proletariat; and there seems reason to believe that, if the St. Petersburg epidemic is of more than common severity, this is only in result of extremely aggravated conditions of privation, overcrowding, filth, and district unwholesomeness, operating on large masses of the lowest population.

"It is probable that the relapsing fever has caused particular alarm in St. Petersburg, from the fact that the disease had never before prevailed there, nor, indeed, been much known anywhere in Russia. But it is a disease greatly less dangerous than typhus. To persons whom they respectively have attacked in this country, typhus has been seven or eight times as fatal as relapsing fever. Both typhus and relapsing fever are communicable from person to person, by means of the general exhalations of the sick; and the danger of such contagion acquires its utmost development when masses of ill-fed population are crowded together in places which are insusceptible of ventilation. It seems probable that the fevers now prevailing at St. Petersburg are not only extensively diffused, but in their respective kinds are of fully average severity.

"The relapsing fever is causing more than its usual proportion of deaths, and is notably attended with those inflammatory swellings which are known by the name of buboes. Although the reports which are before me do not state the relative frequency with which the latter symptom is observed, yet from the fact of being mentioned in a very general report on the epidemic, I infer that it has been more than commonly frequent, and doubtless it was from this circumstance that there arose the rumors of plague. It is therefore requisite to mention that buboes are by no means exclusively characteristic of the plague, that severe typhus in this country is not unfrequently attended by them, and that also in our other forms of fever, they are sometimes, though far less frequently, observed.

"The great importance, however, of the question which is involved in that symptom of buboes was a main reason for your lordship's thinking it desirable to have an English physician's report in all the elements of the St. Petersburg epidemic; and it was therefore that, under your lordship's orders, I instructed Dr. Whitley to proceed to St. Petersburg, for the purpose of making personal examinations in the matter. He arrived at St. Petersburg on Saturday night last; and his telegram, which I received on Tuesday, informs me in positive terms that 'nothing resembling plague has been observed.'

"The disease in North Germany he describes as having no dependence on the Russian fever.

"That disease (the North German) is one of which, hitherto, England has had no general experience. Even in foreign medical literature, mention of it is but comparatively recent, and the knowledge which relates to it is incomplete. It is a febrile nervous affection, of a very painful and very dangerous kind. By us, for practical purposes, it may be regarded as a new disease; but, in truth it has for the last 28 years been prevailing very extensively in successive small epidemics, both in Europe and America, throughout the entire breadth of the north temperate zone."

Dr. Sanderson was instructed to visit those places about the Lower Vistula, where the singular disorder prevails, and he has reported that he finds "no reason for regarding the disease as pesronally communicable;" that he has "met no single instance in which more than one member of the same family has been attacked; nor has there been any diffusion of the disease in any of the hospitals." Mr. Simons sums up his report as follows:—

"From the foregoing statement, your lordship will have gathered that, neither as regards the fevers which are present in St. Petersburg, nor as regards the nervous disease which is occurring in North Germany, are the circumstances such as have, on former occasions, led to the adoption of quarantine by this country; that, as regards the importability of the nervous disease, our danger in communicating with the Baltic ports—unless there were movement of masses of infected population—is apparently nothing, or next to nothing, and that, as regards the Russian epidemic, our danger in communicating with St. Petersburg is only the same sort of danger as the several parts of the United Kingdom have often occasioned to one another, and are even at the present time, as regards the worst known forms of fever, daily and abundantly occasioning.

"As, however, it is possible, and even probable that, with the reopening of the Baltic navigation, ships which come to our port from Russian territory may occasionally have fever cases on board, it is essential that, in places which this danger may affect, the local sanitary authorities should exercise peculiar vigilance against all those unwholesome conditions which favor the propagation of such disease."

